# Improving the power performance of urine-fed microbial fuel cells using PEDOT-PSS modified anodes

**DOI:** 10.1016/j.apenergy.2020.115528

**Published:** 2020-11-15

**Authors:** M.J. Salar-Garcia, F. Montilla, C. Quijada, E. Morallon, I. Ieropoulos

**Affiliations:** a Bristol BioEnergy Centre, Bristol Robotics Laboratory, University of the West of England, Coldharbour Lane, BS16 1QY Bristol, United Kingdom; b Departamento de Química Física e Instituto Universitario de Materiales, Universidad de Alicante, Crtra. San Vicente s/n 03690, E-03080 Alicante, Spain; c Departamento de Ingeniería Textil y Papelera, Universitat Politècnica de València, Pza Ferrandiz y Carbonell, E-03801 Alcoy, Alicante, Spain

**Keywords:** Microbial fuel cells, PEDOT-PSS, Bioenergy, Urine

## Abstract

The need for improving the energy harvesting from Microbial Fuel Cells (MFCs) has boosted the design of new materials in order to increase the power performance of this technology and facilitate its practical application. According to this approach, in this work different poly(3,4-ethylenedioxythiophene)-polystyrenesulfonate (PEDOT-PSS) modified electrodes have been synthesised and evaluated as anodes in urine-fed MFCs. The electrochemical synthesis of PEDOT-PSS was performed by potentiostatic step experiments from aqueous solution at a fixed potential of 1.80 V (*vs*. a reversible hydrogen electrode) for different times: 30, 60, 120 and 240 s. Compared with other methods, this technique allowed us not only to reduce the processing time of the electrodes but also better control of the chemical composition of the deposited polymer and therefore, obtain more efficient polymer films. All modified anodes outperformed the maximum power output by MFCs working with the bare carbon veil electrode but the maximum value was observed when MFCs were working with the PEDOT-PSS based anode obtained after 30 s of electropolymerisation (535.1 µW). This value was 24.3% higher than using the bare carbon veil electrode. Moreover, the functionality of the PEDOT-PSS anodes was reported over 90 days working in continuous mode.

## Highlights

• Successful electropolymerisation of PEDOT-PSS onto carbon veil anodes.
• Up to 24.3% of power improvement when using PEDOT-PSS based anodes (535.1 µW).
• The power output by MFCs is maximum with 6.12 × 10^−6^ g PEDOT cm^−2^ anodes.
• Good functionality of the PEDOT-PSS anodes for 90 days in continuous mode.

## 1 Introduction

Microbial Fuel Cell (MFC) is an environmentally friendly technology, which relies on the ability of electrogenic bacteria to transform the chemical energy stored in a substrate into electricity. The interest in MFCs has significantly increased in the last three decades due to this technology contributing positively towards two of the most concerning environmental challenges: increasing global energy needs and waste treatment [1], [2], [3]. Conventional wastewater treatment techniques are usually energy-consuming and generate waste, which needs to be managed. MFCs are a sustainable technology able to meet the current sanitation needs while producing electricity, reducing the organic content and recovering of nutrients and metals [4].

MFC consists of an anodic compartment where bacteria oxidise the organic matter contained in a simple or complex substrate, releasing protons, electrons and small molecules. The electrons flow from the anode to the cathode through an external circuit. At the cathode an oxidant is reduced, usually the oxygen in the air, completing the redox reaction. Anodic and cathodic chambers are physically separated by a selective membrane whose main functions are not only to maintain the distance between the electrodes to avoid the short-circuiting of the system but also reduce the substrate losses, oxygen diffusion to the anode and diffusion of substrate or products of its oxidation to the cathode.

So far, a wide variety of substrates has been used as feedstock in MFC for bioenergy production and waste treatment, e.g. domestic wastewater, waste stream from food processing industries, beer or paper production industries, urine, etc. [5], [6]. Among them, the use of neat human urine offers several benefits over other wastes. Its natural abundance, moderate pH and buffering capacity, high conductivity and chemical oxygen demand (COD) values have promoted its use in different kinds of bioelectrochemical systems such as MFCs [7], [8], [9]. This energy-rich substrate has been successfully used as the sole feedstock in MFC to generate enough electricity to charge phones or smartphones. In particular, Ieropoulos et al. 2013 [10] reported, for the first time, the use of neat human urine to directly charge the batteries of a mobile phone, which opened up the possibility of transforming this human waste into useful energy, for instance, in remote locations. In 2017, Walter et al. [11] were able to charge a smartphone for 3 h of operation, including outgoing calls, by using 600 mL of neat human urine and 6 h of charging process. Their new design allowed them to overcome the energy losses caused by the battery capacity of commercially available phones (38%). More recently, the same authors employed a cascade of MFCs modules to directly charge a microcomputer. Each cascade of four MFC modules was able to reach a continuous power output of 130 mW, which was enough to directly power the microcomputer requirements [12]. Despite this work demonstrates the potential of MFCs for producing directly exploitable energy levels, the authors also highlighted the importance of the robustness of the system designed for long periods of operation and the stability of the electrical connections, source of energy losses. Another practical application of urine-fed MFCs is their use for lighting. Ieropoulos et al 2016 [13] conducted the first trial on Frenchay Campus (UWE, Bristol) in 2015 where modular urine-fed MFCs demonstrated their feasibility for lighting, generating an average of 75 mW (288 MFCs). Then, the system was scaled-up to be used during Glastonbury Music Festival at Worthy Farm the same year. In this case, the system was able to power for internal lighting from the festival attendants, generating an average of 300 mW (432 MFCs). This field trial not only reported the feasibility of MFCs for generating electricity but also for sanitation since 30% of the chemical oxygen demand (COD) was removal from urine. The MFC design used in this field trial was then improved by Walter et al. in 2018 increasing the power output up to 30% by using 1/3 of the total volumetric footprint [14]. The new design also increased the COD removal compared to the previous field trial, reaching a value of 92% from half the retention time. These results demonstrate the feasibility of MFCs for generating usable electricity from urine, which opens up the opportunity to generate electricity in remote locations or developing areas where the energy infrastructure is poor.

In order to advance MFCs and move closer to practical implementation, it is crucial to work on new materials design and reactor architectures, which will allow us not only to improve the power performance of this technology and its treatment capacity but also reduce its cost [15], [16], [17]. Ceramic materials have emerged as a promising alternative to commercial polymer membranes, e.g. Nafion or Ultrex. The natural abundance, low cost and robustness of this kind of materials promote their use in commercial systems with low environmental impact. Another benefit is that their properties, e.g. porosity, ionic conductivity or water absorption, can be finely tuned by changing the kilning procedure or doping the raw material with other compounds [18], [19], [20], [21], [22]. Regarding the catalyst in the cathode, platinum group metals (PGMs) have been widely used in the past due to their high catalytic activity, however their high cost has boosted the onset of alternative materials [23], [24], [25], [26]. In the case of anode materials, among which the most used are carbon-based materials such as activated carbon because of its low price, good long-term stability and biocompatibility [23].

As microbes are the main drivers of this technology, the development of an efficient biofilm round the anodic electrode is crucial for the well-performing of the system [27]. The anode surface plays an important role in microbial growth and electron transfer processes between electroactive bacteria and the electrode, which directly affect the power performance of the system and also its treatment capacity. To this end, great advances have been made so far in terms of anode materials and anode surface modifications in order to enhance the power performances of MFCs and therefore promoting their practical application. In the past, carbon materials, e.g. graphite rod, graphite felt, carbon cloth, flexible graphite sheets, graphite granules or activated carbon, have been widely used due to their reduced cost, electrical conductivity, chemical stability and biocompatibility. However, in recent years new methods for surface modification have emerged with the aim of designing favourable environments for developing efficient biofilm capable of improving the electron transfer from microbes to the anodic electrode [28], [29], [30], [31].

Among the different type of anodes modification, the use of conducting polymers has gained much attention in recent years due to the increase of the surface area and the acceleration of the electron transfer. Despite its lack of conductivity at neutral pH, polyaniline (PANI) has been widely used in MFCs because of its thermal stability, corrosion resistance and ease of polymerisation [32]. Recently, an electrochemical deposition method was used to elaborate novel anodes made of carbon veil modified with Fe_2_O_3_-polyaniline-dopamine hybrid composite by Jian et al. [33]. These electrodes were used in MFCs to efficiently degrade indole, reaching up to 90.3% in 120 h coupled to an increase in the power output too, compared with the bare electrode. More adequate conducting polymers for biological applications are based in pyrrole or thiophene units due to its good electrocatalytic properties, high conductivity at neutral pH and environmental stability as well as its low cost. Jia et al. [34] compared the efficiency of PANI/multi-walled carbon nanotubes and polypyrrole/multi-walled carbon nanotube composites electrochemically deposited onto graphite rod as anodes for improving the power performance of fluidic bed MFCs. This work reported that the resistance of the anodes containing polypyrrole is lower than those containing PANI, and much lower than the bare graphite rod, which results in higher conductivity of the polypyrrole-based anodes compared to the rest of the materials. These results are reflected not only in the power output by the system but also in its treatment capacity, in terms of COD removal. Poly(3,4-ethylenedioxythiophene) (PEDOT) is one of the most successful conducting polymers in terms of practical applications due to its ability to form thin and homogeneous films, high electrical conductivity, and good physical and chemical stability. For these reasons, PEDOT has gained much attention for being used in solar cell and biosensor technology as well as electrode material [31]. The addition of PSS (poly-styrene sulfonate) acting as a surfactant and doping agent allows the polymerisation with higher concentrations of monomer in solution. The electron transfer mechanism involves PSS units of the conducting polymer composite, enhancing the electron transfer [35]. The need for light and durable supercapacitors for electronic energy storage devices has boosted the search for cost-effective and functional materials easily to synthesise for promoting their practical application. Cho et al. [36] reported that the conductivity of graphene films increased significantly when modified with PEDOT-PSS, which avoid the need to use a metallic current collector. The PEDOT-PSS based supercapacitors showed good electrochemical properties and mechanical stability, facilitating its application in electronic or energy storage devices. This versatile conductive polymer has been successfully used as a transparent anode in organic solar cells either as a transparent electrode [37], [38], a hole transport layer [39] or as part of photoactive composite layers [40] in efficient organic solar cells and polymer light emitting diodes.

In this context, this work explores for the first time, simple, inexpensive and well-controlled electrode modification process by *in situ* electropolymerisation of poly(3,4-ethylenedioxythiophene) doped with poly-(styrenesulfonate) (PEDOT − PSS) for being used as anodes in urine-fed MFCs. This work focuses on not only increase the power performance of MFCs but also improve its stability and functionality for long operating processes, commonly used in real practical applications. The aim is to develop an affordable MFCs design whose power performance does not compromise its practical implementation. To the best author’s knowledge, this is the first time that this type of anode material has been tested in MFCs continuously fed with neat human urine.

## 2 Material and methods

### 2.1 Synthesis of the PEDOT-PSS modified anodes

PEDOT-PSS electropolymerisation was performed with the following precursor solution: first, an aqueous poly(sodium 4-styrenesulfonate) solution (Na-PSS, Aldrich, average M_w_ ~ 70,000, powder) of 146 g.L^−1^ was prepared, then 3,4-ethylendioxythiophene (EDOT, Aldrich, 97%) was added to obtain a concentration of 45 mM of monomer. This mixture was stirred under an ultrasonic field for 30 min [41]. The solutions were prepared with ultrapure water obtained from an Elga Labwater Purelab system (18.2 MΩ cm).

In the present study, PEDOT-PSS modified anodes were synthesised by potentiostatic step experiments at a fixed potential of 1.80 V and at different times: 30, 60, 120 and 240 s. Accordingly, the different anodes synthesised will be referred to as ST-30, ST-60, ST-120 and ST-240 respectively.

Carbon veil (20 g∙m^−2^, PRF composites, UK) was used as the supporting material for the PEDOT-PSS. A piece of 5 × 10 cm^2^ was rinsed with abundant ultrapure water prior to its use in the electrochemical cell. Electrochemical synthesis and characterisation of the anodes were performed with an eDAQ Potentiostat (EA163 model) coupled to EG&G Parc Model 175 wave generator and the data acquisition was performed with eDAQ e-corder 410 unit (Chart and Scope Software). All the potentials were measured against a reversible hydrogen electrode (RHE) immersed in the same electrolyte and are presented in that scale. A platinum wire was used as the counter electrode. The current density was calculated from the geometric area of the working electrode immersed in the solution.

### 2.2 MFC set-up and inoculation

Acrylic air-breathing single-chamber MFCs with an empty volume of 12.5 mL were used to perform the experiments. Cathodes were made of a blend of activated carbon and polytetrafluoroethylene (PTFE) (186 ± 7 mg∙cm^−2^) [42] pressed over a piece of stainless steel (12.25 cm^2^) whereas the anodes consisted of carbon veil of 5 × 10 cm^2^ coated with PEDOT-PSS as previously described. As a membrane, flat pieces of terracotta clay (3.5 × 3.5 cm^2^) kilned at 1030 °C and 7 h of ramp time were used [21]. After kilning, the final thickness of the membranes was 1.5 mm.

The MFCs were inoculated with a mixture of sludge and neat human urine (1:1 v/v) in batch mode. This solution was replenished with a fresh mixture daily for 4 days. Then, the MFCs were continuously fed with neat human urine at a continuous feed flow of 0.06 mL∙min^−1^. During the start-up time, the external resistance was adjusted and finally, it was kept constant at 900 Ω according to previous experiments with similar anode size. The voltage of the system was continuously monitored by a multichannel Agilent recorder data logger (LXI 34972A data acquisition/Switch unit) for 90 days. 4 different PEDOT-PSS modified anodes and the bare carbon veil were tested in triplicate with a total number of 15 MFCs run in parallel.

### 2.3 Electrochemical measurements in MFC

The polarisation of the MFCs using the different PEDOT-PSS modified anodes was performed by linear sweep voltammetry (LSV) (μAutoLab III/FRA2, Metrohm, The Netherlands) from open-circuit voltage (OCV) to 0.05 V at a scan rate of 0.25 mV∙s^−1^. The measurements were performed once reached a stable OCV in a two-electrode configuration where the anode was connected to the counter electrode, the cathode was connected to the working electrode and reference channel short-circuited with the counter electrode channel. Polarisation curves were obtained by plotting the cell voltage *versus* current (V *vs.* I) whereas power curves were obtained by plotting power *versus* current (P *vs*. I).

### 2.4 X-Ray Photoelectron spectroscopy (XPS) and scanning electron microscopy (SEM) analysis

X-Ray Photoelectron spectroscopy (XPS) was conducted in a K-ALPHA spectrometer (ThermoFisher Scientific) by using a microfocused monochromatised Al Kα radiation (1486.6 eV) of 400 μm spot size (3 mA × 12 kV) at a base pressure below 5 × 10^−10^ kPa. Photoelectrons were collected into a hemispherical analyser operated in the constant energy mode at pass energy of 200 eV and 50 eV for survey and narrow core-level spectra. Peak binding energies (BE) were referenced to the principal C1s line at 284.6 eV and given to an accuracy of ± 0.2 eV. Core-level S2p peaks were deconvoluted by using spin–orbit doublets with an splitting gap of 1.1–1.2 eV and an intensity peak ratio of 2:1 [43]. Data were analysed with Thermo Scientific™ Avantage software. A smart correction function was used for background subtraction. Peak synthesis was done with mixed Gaussian (70%)/Lorentzian (30%) function line shapes. Surface charging was compensated with a flood electron gun. The morphology of the modified electrodes was determined by a SEM microscope (Hitachi S3000 N). *ImageJ* software was employed for the analysis of SEM images.

## 3 Results and discussion

### 3.1 Synthesis and characterisation of PEDOT-PSS anodes

**Fig. 1:**
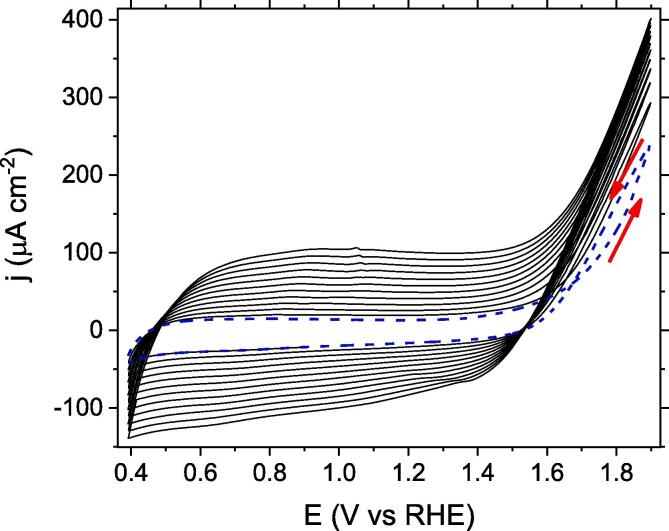
Cyclic voltammogram of a carbon veil electrode in Na-PSS solution (146 g∙L^−1^) containing 45 mM of EDOT. Blue dashed line: First voltammetric scan. Second and successive scans are depicted in black solid lines. Scan rate: 100 mV s^−1^.

Fig. 1 shows the cyclic voltammograms of a carbon veil electrode immersed in the precursor solution for the electropolymerisation of PEDOT-PSS.

The first potential cycle is a featureless voltammetric profile up to a potential of 1.55 V. Above that potential a positive current appears related to the onset of the EDOT monomer oxidation. In the backward scan, the oxidation current density is higher than in the forward scan producing a so-called nucleation loop, which is indicative of the electrodeposition of PEDOT-PSS. The progressive increase in the current plateaus between 0.4 and 1.4 V observed during the second and following scans up to 1.9 V confirms the growth of a conducting polymer PEDOT-PSS film on the carbon veil electrode as a result of the monomer oxidation.

Once established that the oxidation from 1.55 V leads to the polymer formation, the electrochemical synthesis of PEDOT-PSS was performed by potentiostatic step experiments at a fixed potential of 1.80 V.

**Fig. 2:**
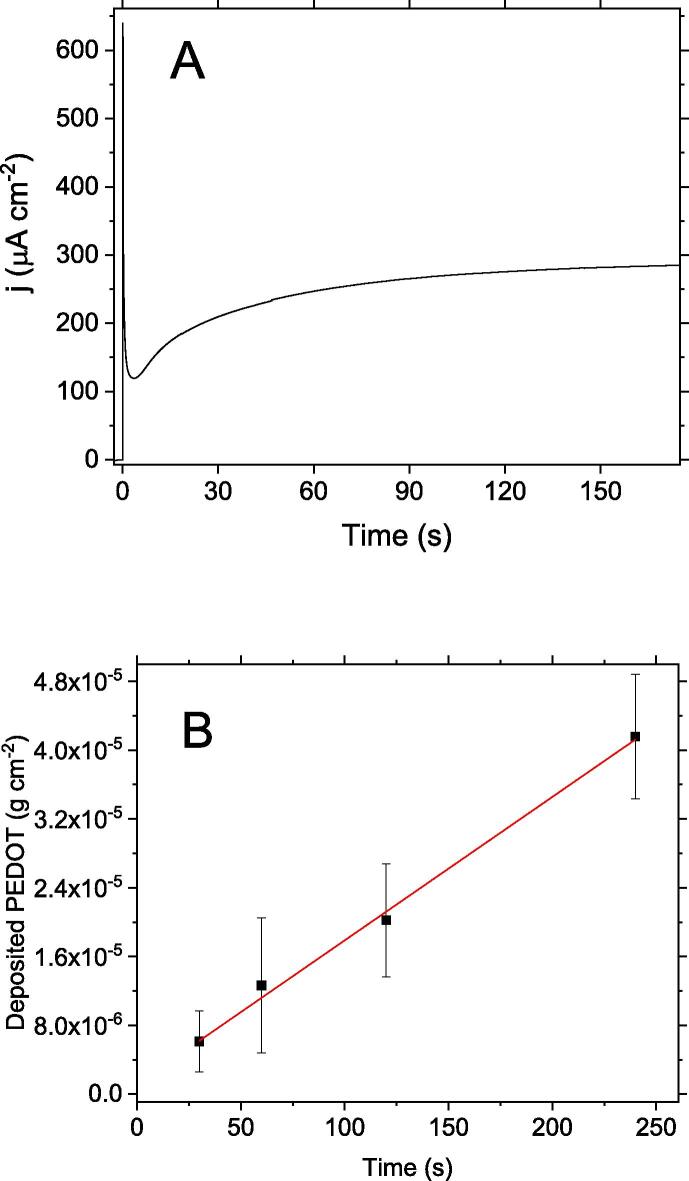
(A) Chronoamperogram of a carbon veil electrode in Na-PSS solution (146 g∙L^−1^) containing 45 mM of EDOT. Initial potential 0.40 V. Final potential: 1.80 V. Fig. (B) Amount of PEDOT deposited on carbon veil electrode as a function of the time of the potentiostatic step at 1.8 V. For each experimental condition, at least 4 electrodes were prepared. Error bars indicated the standard deviation of each measurement.

Fig. 2A shows a typical chronoamperometric experiment for a carbon veil electrode in the polymer precursor solution.

The general feature of the chronoamperogram is comparable with the curves reported in literature for EDOT and related thiophene monomer oxidation [44]. After the double layer charging, the current–time transient observed is characteristic of an instantaneous nucleation mechanism for the polymerisation with a 3D growth under charge transfer control [45]. This type of mechanism drives to uniform deposition of the polymer on the electrode surface.

**Fig. 3:**
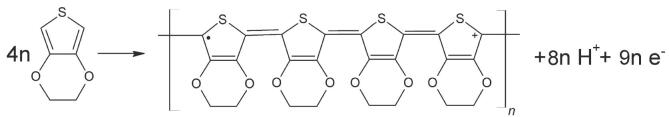
Electrochemical reaction for the electropolymerisation of PEDOT.

The amount of deposited electroactive polymer can be estimated from the faradic charge of the chronoamperometric experiment by assuming a charge of 2.25 e per EDOT monomer units deposited, assuming the formation of a doped conjugated polymer with one polaronic moiety in four monomer unit domains (see Fig. 3).

Fig. 2B shows the amount of deposited PEDOT as a function of the different time for the potentiostatic step, 30, 60, 180 and 240 s. A linear trend is observed showing a deposition rate of PEDOT of 0.17 µg∙s^−1^. It should be noted that the mass calculated so far most likely underestimates the real mass of the polymer deposited. Since PEDOT present positive polaronic charges, these charges must be compensated with anionic PSS units giving rise to PEDOT-PSS. Doping PSS chains remain entrapped within the polymer film, thereby leading to an obvious increase in weight over that of the conjugated polymer fraction. Further details on the real mass and composition of the films will be given below.

**Fig. 4:**
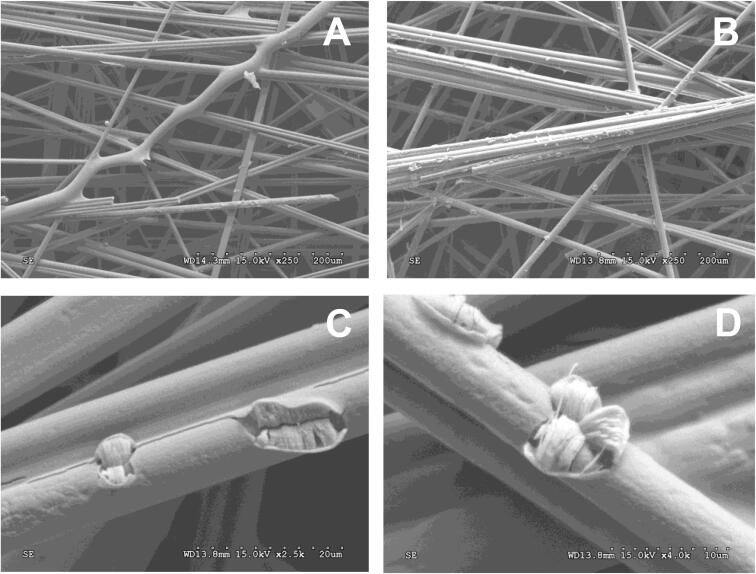
SEM images for bare carbon veil electrode (A) and PEDOT-PSS-modified electrodes, ST-60 (B, C and D).

After the electrochemical polymerisation, the electrodes were withdrawn from the cell and rinsed with ultrapure water for their characterisation by SEM, XPS and cyclic voltammetry. Fig. 4 shows SEM images for bare carbon veil and a PEDOT-PSS-modified electrode (ST-60) as an example of the growth of the conducting polymer around the carbon fibres of the supporting material.

As observed the bare carbon veil is composed of uniform fibres (see Fig. 4A, average diameter of 11.2 µm). After the deposition of PEDOT-PSS (see Fig. 4B) the morphology of the samples seems unaffected, but the diameter of the fibres seems higher (average diameter of 11.5 µm) indicating that the PEDOT is being uniformly deposited around the carbon fibres. However, in a closer view of the modified electrode (Fig. 4C and 4D) some areas of the material appear cracked, thus revealing the occurrence of a uniform polymer film around the carbon fibres, as well as its inner aspect. In previous work, we also reported the deposition of smooth and featureless PANI thin films on activated carbon cloths by galvanostatic electropolymerisation at short time [46]. The inner strands of PEDOT-PSS present diameters of about 250–300 nm. Little differences were observed between the different PEDOT-PSS modified electrodes.

**Fig. 5:**
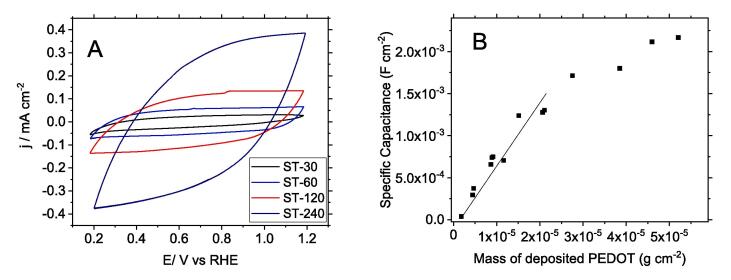
(A) Stabilised cyclic voltammogram of carbon veil electrode modified with PEDOT-PSS (ST-60) in 0.146 g∙mL^−1^ of poly(sodium 4-styrenesulfonate) (Na-PSS). Scan rate: 100 mV∙s^−1^. Fig. (B) Specific capacitance of PEDOT-PSS films determined from voltammogram against the amount of electrodeposited PEDOT.

PEDOT-PSS-modified electrodes were characterised by cyclic voltammetry in a blank solution of Na-PSS. Fig. 5 shows the voltammogram of different electrodes, which are essentially featureless showing a capacitive character in the whole range of potentials scanned.

The most remarkable differences are related to the current density that is proportional to the amount of conducting polymer deposited. The specific capacitance referred to the geometrical area of each electrode synthesised can be determined from the voltammetric experiments by means of Eq. (1):

(1) $$C=\frac{\Delta q}{\Delta V}$$

being Δq the specific charge (C cm^−2^) in a potential interval of ΔV. The specific capacitance is plotted as a function of the electrodeposited mass of PEDOT in Fig. 5B. As observed, this parameter increases linearly with the amount of polymer up to values around 20 µg∙cm^−2^. From the slope of this plot a value of mass capacitance 82F∙g^−1^ PEDOT is obtained, which is similar to the values found in the literature [47]. For higher values of deposited PEDOT the curve flattens, and the specific capacitance tends to level off. The worsening of the electrochemical properties of thick PEDOT-PSS films was previously observed for electron transfer to cyt c [41], [48]. In that work it was shown a decline of electrocatalytic properties for amounts of deposited PEDOT higher than 20 µg∙cm^−2^ (equivalent to a thickness of 0.2 µm), attributed to a nonuniform vertical conductivity, probably due to the existence of a vertical conductivity gradient at PEDOT-PSS.

**Fig. 6:**
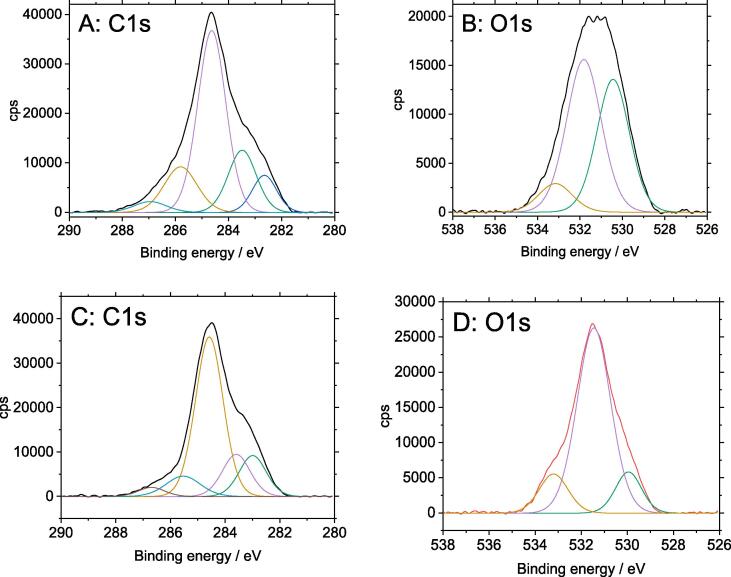
High resolution X-ray photoelectron spectra for C1s and O1s transitions for bare carbon veil (A and B) and PEDOT-PSS films (C and D).

XPS spectroscopy was used to provide information on the state of bonding and local atomic environment of C, O and S photoelectrons of surface groups on PEDOT-PSS-modified samples, so to shed some light on the elemental composition of these composite electrodes. High-resolution spectra of C1s and O1s core-level photoelectron transitions are shown in Fig. 6. The spectral line shapes and curve fittings are qualitatively identical for composites obtained at different electropolymerisation times. Spectra for the bare carbon veil support are also included for comparison.

The C1s core-level spectrum for the carbon veil (Fig. 6A) can be fitted with up to five different peak components. The major peak at 284.6 eV corresponds to aromatic carbons in graphene layers and the higher BE contributions arise from minor amounts of alcohol/ether (285.8 eV) and carbonyl (287.0 eV) surface groups. The contributions below 284 eV are known to involve carbidic structures [49]. The C1s spectrum for PEDOT-PSS-modified electrode (Fig. 6C) still shows the distinctive carbide signals from the underlying support. Therefore, it is expected that the peak components above 284 eV have shared contributions from the support and from skeletal C-C/C = C in PSS, C-S structures in both PEDOT and PSS, and PEDOT dioxyethylene carbons [50].

The O1s core-level line of the PEDOT-PSS films displays a principal peak component at 531.5 eV and a minor peak at 533.2 eV. These photoelectron signals are associated with oxygen in sulfonate groups from PSS and dioxyethylene groups from PEDOT [50]. These spectral features can be partly contributed to by O1s core-level photoelectrons from the support. The third minor peak component at about 530 eV entirely corresponds to oxygen-containing surface groups from the carbon veil, probably of the keto/quinone type [51].

**Fig. 7:**
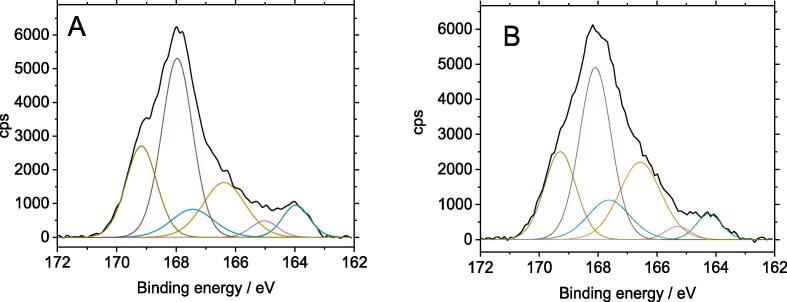
High resolution S2p core-level photoelectron spectra of PEDOT-PSS under potentiostatic control (A) ST-60 and (B) ST-240 samples.

Fig. 7 shows the S2p core-level photoemission lines for two PEDOT-PSS films. It reflects the chemical state of sulphur-containing molecular structures belonging exclusively to the polymer fraction of the modified electrodes.

The XP spectrum of the modified electrode shows three different spin–orbit doublets with their S2p_3/2_ component located at 164.0, 166.4 and 168.0 eV. The contributions at 164 and 168 eV were reported for PEDOT-PSS films electrosynthesised potentiodynamically on Au, ITO and carbon glassy electrodes [41], [50] or spin-coated from commercial PEDOT:PSS suspensions on a variety of substrates [52], [53], [54]. These signals are assigned to thiophene ring S in EDOT units and sulfonate groups in PSS chains respectively. PEDOT-PSS modified electrodes show an additional S structure of intermediate binding energy, which may be ascribed to thiophene S-oxide subunits in EDOT segments. The chemical state of these S-oxide structures is intermediate between that of aromatic sulfoxides (>S = O, 165.2 eV) and sulfones (>SO_2_, 167.8 eV) [55], [56]. The relative surface abundance of the different sulphur atomic environments identified on the PEDOT-PSS-modified electrodes can be calculated from the area ratio of sulfonate peak components to thiophene peak components (including neutral and thiophene S-oxide units). In the present case the monomer styrenesulfonate:ethylenedioxythiophene (SS:EDOT) ratio in the polymer was around 1.6 for these electrodes. The excess of PSS indicates that the surface of the polymer modified electrodes is negatively charged even in the doped state. With this atomic ratio we can determine that the real mass of PEDOT-PSS deposited polymer is around 3.4 times higher than that determined from the charge of chronoamperometric experiments (where it was assumed that only EDOT monomers were deposited). Therefore, the PEDOT-PSS mass deposited for a ST-60 electrode should be 43 µg∙cm^−1^, which gives a polymer thickness of about 0.4 µm if one assumes a polymer density of 1 g∙cm^−3^ [41]. This value agrees with the estimation from SEM images of Fig. 4.

The value of the SS:EDOT ratio of the present electrodes contrasts with the composition of films grown by cyclic voltammetry, where the ratio was close to 4 [41], [50]. It suggests a strong depletion in the PSS content of PEDOT-PSS formed on carbon veil substrates. In these samples, the removal of excess insulating polyelectrolyte ions may lead to a more efficient separation between PEDOT conjugated system and PSS chains, thus allowing the formation of more conductive PEDOT channels that enhances the conductivity of the composite and offsets the oxidation of some thiophene units.

### 3.2 Microbial fuel cell performance

**Fig. 8:**
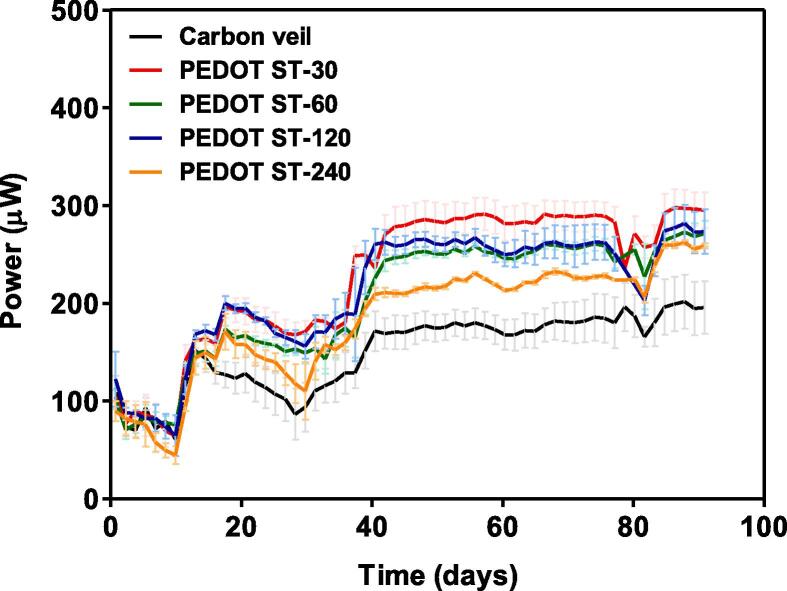
Long-term power performance of the MFCs working with the different anodes synthesised.

The long-term stability of MFCs is one of the most important parameters for the practical application of this technology. In this work, the MFCs were running for 90 days (3 months) continuously fed with neat human urine. Fig. 8 depicts the evolution of the average power output over time for each type of anode assessed in triplicate. After the maturing stage, the power output by the systems remained stable, regardless of the type of anode used, which means that the materials used for the MFCs set-up are suitable for long-term working periods. Around day 80 there was a lack of substrate which matches with the reduction in power observed. However, once the feeding problems were fixed, the power output by the systems went back to the previous stable value. As can be seen, the presence of PEDOT-PSS over the anode surface has a significant effect on the power output by the MFCs. Regardless of the electropolymerisation conditions, all PEDOT-PSS modified anodes outperformed the bare carbon veil anode. Among them, the PEDOT-PSS modified anode obtained after 30 s of potentiostatic step (ST-30, estimated polymer thickness of 0.21 µm) offers the highest value of average stable power (283.5 µW). Despite all PEDOT-PSS modified anodes exhibit higher values of continuous power output than the bare carbon veil, it was observed that thicker PEDOT-PSS films reduce the effect of the conductive polymer on the power performance of the MFCs. In the case of the ST-60 and ST-120 anodes (with estimated polymer thickness of 0.43 and 0.69 µm, respectively), they were able to reach similar values of stable power output (252.1 and 259.9 µW, respectively). Finally, the ST-240 anode (estimated polymer thickness of 1.41 µm) allowed the MFCs to reach a stable power output value lower than the rest of modified anodes (220.8 µW) but still higher than the value observed when the MFCs are working with the bare carbon veil anode (188.4 µW).

**Fig. 9:**
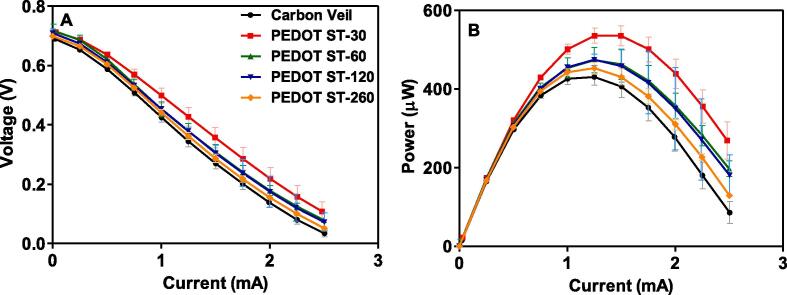
Polarisation (A) and Power (B) curves of the MFCs working with the different anodes synthesised.

Fig. 9A and B show the polarisation and power curves of the MFCs when they reached the steady-state, respectively. According to the long-term power output results showed in Fig. 8, the overall polarisation and power curves exhibit similar trend. The maximum power output by the MFCs working with all PEDOT-PSS modified anodes outperformed the systems working with bare carbon veil. The highest value of power output was observed in the case of the modified anode elaborated with a 30-s potential step (535.1 µW), 24.3% higher than using the bare electrode. The systems working with the modified anodes synthesised for 60 (ST-60) and 120 s (ST-120) steps showed very similar values of maximum power (473.3 and 474.3 µW, respectively) but lower than ST-30 anode. The case of the ST-240 anode reached the lowest value of maximum power output compared to the modified anodes (452.6 µW). However, this value was still 5.1% higher than that reached by the system working with the bare carbon veil anode (430.5 µW). The results show that despite all PEDOT-PSS modified anodes outperformed the bare anode, the deposition of films thicker than 0.2 µm negatively affects the power performance by the MFC set-up investigated. The longest the electropolymerisation time of the conductive polymer onto the carbon veil, the highest the amount of PEDOT-PSS electrodeposited on the surface of the supporting material (see Table 1). High amounts of PEDOT-PSS might increase the internal resistance of the anode and therefore, reduce the power output by the systems [57]. The amount of PEDOT-PSS electrodeposited at the longest electropolymerisation time (240 s) is more than 6 times higher than the amount electrodeposited at the shortest time (30 s) (see Table 1). These results agree well with the studies of direct electrochemistry of cyt c in solution with PEDOT-PSS modified electrodes, in which optimal electrocatalytic performances were obtained from conducting polymers with thicknesses around 0.1–0.3 µm [41].

**Table 1:** Summary of the amount of PEDOT-PSS deposited onto the anode surface, PEDOT-PSS film thickness and maximum overall and normalised power output.

Anodes	Max. Power (µw)	Max. Power normalised to area of anode (µW∙cm^−2^)	PEDOT deposited (g∙cm^−2^_anode_)	PEDOT-PSS thickness (µm)	Max. Power normalised to amount of PEDOT-PSS deposited (W∙g^−1^)
Carbon Veil	430.5	8.61	–	–	–
ST-30	535.1	10.70	6.1 × 10^−6^	0.21	1.75
ST-60	473.3	9.47	1.3 × 10^−5^	0.43	0.73
ST-120	474.3	9.49	2.0 × 10^−5^	0.69	0.47
ST-240	452.6	9.05	4.2 × 10^−5^	1.41	0.22

Liu et al. obtained a power output improvement of 43% by using carbon cloth anodes modified with PEDOT [58]. In this case, the conducting polymer was electrochemically polymerised using a galvanostatic method at a constant current density of 200 μA∙cm^−2^ during 10 min. The synthesis was performed in aqueous media without surfactant which might cause the overoxidation of the polymer during the synthesis because of the low solubility of the monomer. Moreover, this galvanostatic method could have also oxidised the carbon cloth surface and therefore, modified its surface chemistry. By contrast, the operating conditions of the electropolimerisation method employed in the present work were milder than those used by Liu et al. which allowed us to better control the polymerisation process and avoid the chemical modification of the supporting electrode. In this case, the XPS results confirmed the proper composition of the polymer synthesised and the absence of defects in its structure, which show the efficiency of the technique. In addition to the better control of the chemical properties of the deposited polymer, the method used in the present work also reduced the electropolymerisation time compared with the galvanostatic technique used by Liu et al., which allowed us to reach 24.3% of power improvement by using the anodes electropolymerised for 30 s. Along with the electropolimerisation method, there are also significant differences between the MFC set-up and inoculation method of the present work and that reported by Liu et al. which might explain the differences in terms of power output. In their case, the double chamber MFCs were inoculated with a pure culture of *Shewanella Ioihica* fed in batch mode with phosphate-buffered saline supplemented with lactate whereas we used activated sludge as inoculum and real waste as a substrate for energy production.

A different method was used by Kang et al. to elaborate PEDOT modified anodes [59]. In this case, the authors chemically synthesised the conductive material and then deposited onto the surface of three different carbon-based supporting materials such as graphite plate, graphite felt and carbon cloth. The graphite felt was dipped into a PEDOT-based solution whereas the surface of the graphite plate and the carbon cloth was coated by applying a paste containing the required amount of PEDOT and Nafion as a binder. In the case of carbon cloth as supporting material, the PEDOT modified anodes doubled the power output reached by MFCs when working with the unmodified anode (0.79 W∙cm^−2^ and 0.35 W∙cm^−2^, respectively). However, the SEM images of the electrodes showed the presence of agglomerated particles onto the anode surface, which might be caused by the high amount of polymer deposited. These results show the low efficiency of the polymer film deposited and the poor dispersion onto the electrode surface compared with that electropolymerised in the present work. In addition, it might be noted that the differences in power with the present work could be also related to different MFC set-up. Kang et al. used a double chamber MFC with an anodic volume of 150 mL, 12 times higher than the volume used in the present work. Despite the system was inoculated with anaerobic pond sludge, then the MFCs were fed with phosphate buffer, sodium acetate trihydrate and trace elements in batch mode whereas the systems investigated in the current work were continuously fed with neat human urine. The PEDOT modified anodes synthesised by Kang et al. showed good stability and reproducibility during 3 cycles of 8 days each one. These results are in line with those reported in this work since the PEDOT-PSS modified anodes showed good functionality over 90 days working in continuous mode with real waste. The presence of PSS in the anodes improves the stability of the PEDOT in water, which also benefits the long-term stability of the anodes as can be seen in Fig. 8 [60].

The same group of authors investigated the optimum amount of PEDOT, chemically synthesised, coating graphite felt anodes to maximise the power output by similar MFC set-up [57]. Among the different amounts of conductive polymer investigated (2.5, 5.0 and 7.5 mg.cm^−2^), their results showed that the optimum amount of PEDOT to maximise the performance of their systems is 2.5 mg∙cm^−2^. They observed a reduction in the charge-transfer resistance (R_ct_) of the anode containing 2.5 mg∙cm^−2^ of PEDOT compared to the unmodified anode. The addition of 5.0 mg∙cm^−2^ to the carbon felt anode also reduce the R_ct_ compared to the previous one. However, it was also observed that amounts of PEDOT higher than 5.0 mg.cm^−2^ increased the R_ct_ of the modified anode. In this case, the anode containing 7.5 mg∙cm^−2^ of PEDOT doubled the R_ct_ of the anode containing 5.0 mg∙cm^−2^ of the conductive polymer. In terms of power output, despite all PEDOT modified anodes outperformed the bare graphite felt electrode, there were not observed major differences in terms of maximum power output related to the amount of PEDOT presents in the anode. These results are in line with those reported in this work since high amounts of PEDOT-PSS caused by longer electropolymerisation times might increase the charge-transfer resistance, which results in a reduction of the power output by the MFCs.

More recently, stainless steel has also been used as supporting material for PEDOT electrochemically polymerised under potentiostatic conditions (2.5 V during 10 min) [61]. The MFC set-up used by Ma et al consisted of a single chamber membrane-less cylindrical reactor using platinum as a catalyst. The systems were inoculated with fresh anaerobic sludge from a fruit processing industry and then fed with phosphate buffer and sodium acetate in batch mode. The successful electrodeposition of the conducting polymer onto the stainless surface increased its surface area and made it more porous, which increased the electrode capacity. As a result, the MFCs using the modified anodes were able to reach values of power output 6 times higher than the unmodified anode (6.086 mW∙cm^−2^ and 1.019 mW∙cm^−2^, respectively). The R_ct_ of the PEDOT modified anodes was smaller than the bare stainless steel anode, which indicates a reduction in the electrode impedance and also an efficient electron transfer. However, the use of high potential for the electropolymerisation along with aqueous media might have caused the oxidation of the iron electrode surface. In this case, the improvement of the electrochemical properties of the electrode might be caused by the modification of the supporting material surface instead or by the polymer deposited.

Regardless of the synthesis process, all the work previously discussed demonstrate the benefit of using PEDOT modified electrodes as anodes in MFCs. It worth highlighting that the potentiostatic method used in the present work for synthesising the PEDOT-PSS modified anodes offers several benefits over those discussed above. On the one hand, the potentiostatic method used allowed us a better chemical control of the electrodeposited films and therefore, avoid the modification of the carbon veil surface, which confirm that the improvement of the electrochemical properties of the anodes are due to the presence of the conductive polymer and not because of the modification of the surface properties of the supporting material. Thus, the XPS spectra confirmed the proper composition of the conductive polymer films and the absence of structural defects, which reported the efficiency of the method employed. On the other hand, this method also shows a significant reduction of the time needed for the electropolymerisation and the polymer loading required to bring about an enhanced response, which brings an advantage over other synthesis methods. This technique allowed us to elaborate stable anodes which not only improve the power performance of MFCs but also were able to continuously produce electricity for 90 days.

## 4 Conclusions

The aim of this work is to analyse the functionality of PEDOT-PSS modified electrodes as efficient and stable anodes in urine-fed MFCs. To this end, different PEDOT-PSS anodes were synthesised by electropolymerisation onto the surface of a piece of carbon veil under potentiostatic conditions at a fixed potential of 1.80 V and at different times (30, 60, 120 and 240 s). The main benefit of this method is the high chemical control of the electrodeposited polymer films and the significant reduction of the time needed for the electropolymerisation, providing room to improve the anodes. The results showed that all PEDOT-PSS modified anodes outperformed the bare carbon veil electrode, which indicates the improvement of the conductive properties of the carbon veil for the biofilm growth and facilitates the electron transfer. Among the different time studied, the maximum power output was observed for the shortest electropolymerisation time, 30 s. In this case, the maximum power output by the MFCs was (535.1 µW), 24.3% higher than using the bare carbon veil electrode. Moreover, the functionality of the synthesised anodes was evaluated for 90 days where the systems were continuously fed with neat human urine. The performance of the MFCs was stable during this period which demonstrates that the PEDOT-PSS was successfully deposited onto the surface of the electrodes and kept attached there with its electron-transfer properties unchanged during the whole experiment. These results confirm that the use of PEDOT-PSS modified anodes not only improve the electrochemical properties of these electrodes but also facilitate the biofilm growth and therefore, the enhancement of the overall power performance and the long term functionality of the MFCs. Advances in new materials design are crucial for the development of this environmentally friendly technology and further progress towards its practical application.

## CRediT authorship contribution statement

**M.J. Salar-Garcia:** Conceptualization, Data curation, Formal analysis, Investigation, Methodology, Visualization, Writing - original draft, Writing - review & editing. **F. Montilla:** Conceptualization, Methodology, Formal analysis, Investigation, Resources, Writing - original draft, Writing - review & editing. **C. Quijada:** Formal analysis, Writing - original draft, Writing - review & editing. **E. Morallon:** Funding acquisition, Supervision, Writing - original draft, Writing - review & editing. **I. Ieropoulos:** Conceptualization, Funding acquisition, Project administration, Resources, Supervision, Writing - original draft, Writing - review & editing.

## Declaration of Competing Interest

The authors declare that they have no known competing financial interests or personal relationships that could have appeared to influence the work reported in this paper.
